# Supplementary figures and images for: A Novel Symbiotic Formulation Reduces Obesity and Concomitant Metabolic Syndrome in Rats by Raising the Relative Abundance of Blautia
Source: Nutrients. 2023 Feb 14;15(4):956. doi: 10.3390/nu15040956 (PMC9960556; doi:10.3390/nu15040956)

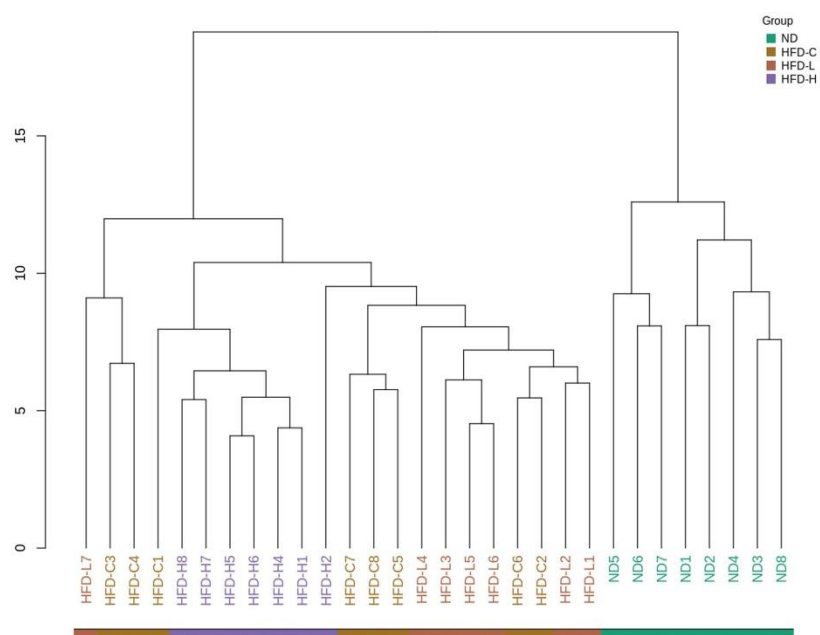

**FigureS3.** Tree analysis of all samples.

Supplement: Supplementary file 1 [file nutrients-15-00956-s001.zip › Supplementary Materials/Supplementary Figures.pdf]
